# UNDERGRAD AND GRAD COMBINED PROGRAMS: FACILITATING RECRUITMENT THROUGH NEW PATHWAYS

**DOI:** 10.1093/geroni/igad104.0637

**Published:** 2023-12-21

**Authors:** Tina Newsham, Elizabeth Fugate-Whitlock

**Affiliations:** University of North Carolina Wilmington, Wilmington, North Carolina, United States; University of North Carolina Wilmington, Wilmington, North Carolina, United States

## Abstract

The University of North Carolina Wilmington was reclassified as an R2 institution in 2018. As such, additional focus has been placed on graduate programs and the generation of research. More recently, UNCW’s Chancellor Volety followed recommendations from faculty, staff, and students to seek to endorse the Age Friendly University principles. These two accomplishments create ideal conditions for growing the gerontology program, which had been on the low productivity list and considered for dissolution (as has happened with many programs around the country). To facilitate recruitment into the MS in Applied Gerontology, new pathways known as “4 + 1” or “combined programs” were created. By allowing qualified undergraduate students to take a portion of their coursework at the graduate level, students explore their interest in gerontology, become invested in gerontology education, and meet undergrad and graduate requirements simultaneously. Students are then able to complete their MS in Applied Gerontology in as little as one more year. Such programming has created a steady stream of students into the graduate program, which is no longer on the low productivity list. While this approach has many strengths, challenges exist that must be acknowledged. Marketing the program to a now very broad prospective student base requires finesse. For example, marketing that would appeal to an exercise science student may not be what speaks to a psychology student. These challenges also translate to the classroom. Although gerontology is an interdisciplinary field, career goals for students now vary even more widely as do their expectations and academic needs.

